# Promoting collagen synthesis: a viable strategy to combat skin ageing

**DOI:** 10.1080/14756366.2025.2488821

**Published:** 2025-04-11

**Authors:** Shan Wang, Feifan Li, Xilong Feng, Meiling Feng, Xiaotian Niu, Xiaoying Jiang, Wenchao Chen, Renren Bai

**Affiliations:** ^a^ School of Pharmacy, Hangzhou Normal University, Hangzhou, Zhejiang, PR China; ^b^ Key Laboratory of Elemene Class Anti-Cancer Chinese Medicines, Engineering Laboratory of Development and Application of Traditional Chinese Medicines, Collaborative Innovation Center of Traditional Chinese Medicines of Zhejiang Province, Hangzhou Normal University, Hangzhou, Zhejiang, PR China

**Keywords:** Collagen, skin ageing, natural product, PPARδ agonist, drug discovery

## Abstract

Skin ageing is a complex physiological process primarily characterised by the deepening of wrinkles and the sagging of the skin. Collagen is essential for maintaining skin elasticity and firmness. As skin ages, it experiences structural and functional changes in collagen, including a decrease in collagen synthesis and an increase in collagen hydrolysis. Thus, promoting collagen synthesis represents a practical strategy for mitigating skin ageing. This review systematically described the functions, classifications and biosynthesis process of collagen, as well as its role in skin ageing. Additionally, the major signalling pathways and targets associated with collagen synthesis were also discussed. More importantly, the review provided a detailed summary of natural products with collagen synthesis-promoting effects and highlighted small molecule compounds with potential anti-ageing activity, especially PPARδ agonists. The relevant content offers potential targets and lead compounds for the development of anti-skin ageing therapies.

## Graphical Abstract

**Figure:**
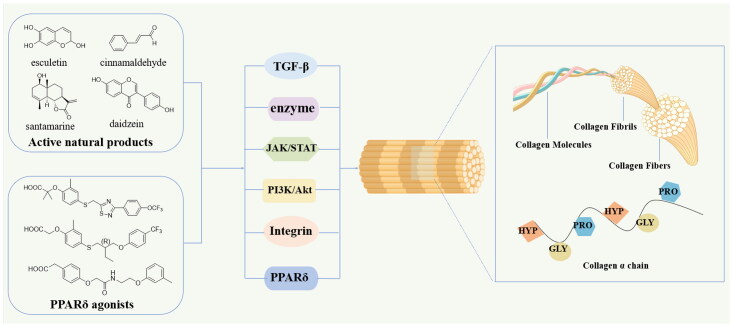

## Introduction

Skin ageing is a physiological process characterised by the deepening of wrinkles, as well as skin laxity and collapse. This process can be categorised into two distinct types: intrinsic ageing and photoageing^1^. Intrinsic ageing refers to ageing that occurs as a result of genetic factors and natural physiological processes, independent of external environmental influences. Increasing with age, the renewal of skin cells decreases and collagen and elastin fibres become insufficient. Intrinsic ageing skin is clinically characterised by a smooth, pale appearance accompanied by fine wrinkles^2^. Photoageing, in contrast, refers to skin ageing induced by ultraviolet (UV) radiation. Prolonged exposure of skin to UV light results in increased levels of reactive oxygen species within the skin tissue, subsequently promoting the overexpression of matrix metalloproteinases (MMPs) under disrupting various signalling pathways. This process leads to enhanced collagen hydrolysis in the skin and ultimately contributes to accelerated skin ageing^3^^,^^4^. Photoageing results in rough, wrinkled skin characterised by hyperpigmentation and capillary dilation^5–7^.

Over time, skin ageing becomes an inevitable process, with one of its hallmark characteristics being the gradual reduction of collagen^8^^,^^9^. As skin ages, collagen undergoes substantial structural and functional changes. Its triple-helix structure breaks down and unwinds, leading to disorganisation within the collagen fibres. This results in a loss of skin elasticity, firmness and smoothness. There is an observed increase in the cross-linking of collagen, leading to greater rigidity and hydrophobicity. This process contributes to the development of dry skin and the formation of deeper wrinkles^10–13^. Research has demonstrated that the reduction of collagen in ageing skin is partially attributable to the role of MMPs in collagen hydrolysis. Overactivation of MMPs can exacerbate collagen breakdown, leading to skin laxity and wrinkles formation^14–16^. Additionally, excessive UV irradiation can cause significant changes in the connective tissue, such as a decrease in the quantity of dermal fibroblasts and a diminished ability for collagen synthesis and alterations in the elastic fibre network, causing curling, relaxation and loss of elasticity^17^. Furthermore, *in vivo* studies indicate that the downregulation of the transforming growth factor-beta (TGF-β) pathway contributes to the decline in collagen expression observed^18^.

Collagen constitutes approximately 30% of the body’s protein composition and serves as the primary protein in the skin, blood vessel, tendon and other connective tissues. It is essential for preserving skin elasticity and firmness^19–21^. Skin ageing is primarily attributed to a reduction in collagen type I content^22^. Thus, promoting collagen synthesis may delay skin ageing.

This strategy of enhancing collagen production is the primary focus of this review. Research has indicated that collagen metabolism is regulated by various signalling pathways, including phosphoinositide 3-kinase/Akt (PI3K/Akt), TGF-β, the Janus kinase/signal transducer and activator of transcription (JAK/STAT) related pathways and peroxisome proliferator-activated receptors beta/delta (PPARβ/δ). Among others, TGF-β functions as a pivotal regulator of extracellular matrix (EMC) components, including collagen and elastin^23–26^. The downregulation of TGF-β levels can inhibit collagen synthesis, contributing to skin ageing. Therefore, promoting collagen synthesis through the modulation of relevant signalling pathways may help delay skin ageing.

This review introduced various strategies to mitigate skin ageing by promoting collagen synthesis. It begins by outlining the critical role of collagen in the skin ageing process, including its functions, classifications and biosynthesis. Subsequently, specific targets and biological pathways that can be harnessed to enhance collagen synthesis and delay skin ageing were discussed. Finally, this review summarised the recent research progress on natural products and small molecule compounds demonstrating potential effects for promoting collagen synthesis.

## Collagen and skin ageing

### Classification of collagen

Collagen is a biopolymer composed of various isoforms that exhibit a triple-helical structure, making it the primary component of the EMC^27^^,^^28^. It is a key structural protein found in animal connective tissues, such as skin, bones, tendons, cartilage and ligaments, playing a vital role in preserving strength and elasticity^29^. Additionally, collagen is essential for joint health, promotes wound healing and supports the integrity of healthy skin. Collagen biopolymers spontaneously assemble into ordered microfibrils and microfibril bundles *in vivo*, creating macroscopic networks and spatial support structures characterised by specific geometries. This process is facilitated by intermolecular recognition^30^^,^^31^.

Collagen can be categorised into six primary types based on its function and structure, numbered with Roman numerals according to the sequence of their discovery: protofibrillar collagen, basement membrane collagen, short-chain collagen, protofibrillar-associated collagen, type XIII and type VI collagen. Protofibrillar collagen generally includes types I, II, III, V and XI. Basement membrane collagen comprises type IV collagen, laminin, heparan sulphate proteoglycans and type VII collagen. Short-chain collagen consists of types VIII and X. Protofibrillar-associated collagen includes types IX, XII and XIV. Type XIII collagen possesses both triple helical and non-collagenous structural domains. Additionally, type VI collagen is a molecule that forms a network and is found in a wide range of tissues throughout the body^32–34^.

The most prevalent types of collagens in living organisms are types I, II and III. In the skin, the predominant collagen is type I collagen, constituting 80% to 90% of the total collagen content^35^. Furthermore, it is the principal collagen found in various connective tissues, including skin, tendons and ligaments, as well as the brain and hyaline tissues. It is primarily synthesised by fibroblasts and is predominantly distributed in the extracellular interstitium, particularly within the dermis. In addition to type I collagen, fibroblasts also produce elastin, a protein that imparts stretching flexibility to the skin by facilitating long-term deformability. The characteristic recoil of elastin aids in restoring tissues to their original shape, which is essential for maintaining skin elasticity^36^. Type II collagen constitutes over 50% of the total protein content in cartilage and accounts for 85% to 90% of the collagen found in articular cartilage^29^. Type III collagen is an EMC protein synthesised by cells into procollagen, which serves as a major structural component of hollow organs, including large blood vessels, the uterus and the intestine^37^. Normal type I collagen is a heterotrimeric, triple-helical linear molecule composed of two α1 chains and one α2 chain. In contrast, types II and III collagen consist of three identical α chains, forming a collagen molecule characterised by a long triple-helical structural domain^38^^,^^39^.

### Structures of collagen

Collagen molecules are categorised into two types: homotrimers and heterotrimers. A heterotrimer, including type I collagen, consists of two α1 polypeptide chains and one α2 polypeptide chain. Conversely, a homotrimer is formed from three α1 polypeptide chains, exemplified by collagen types II and III^40^. Each α polypeptide chain exhibits a left-handed helical structure, and these chains intertwine and are held together by hydrogen bonds, ultimately resulting in the characteristic right-handed helical structure of collagen^41^. Structural proteins typically exhibit more than three levels of organisation, correlating with their physiological functions. In contrast, collagen exhibits a complete four-tier spatial structure, contributing to its unique functional properties^42^.

The arrangement of amino acids in polypeptide chains constitutes the primary structure of collagen at its most fundamental level. Collagen comprises not only non-fibrillar types but also features a repeating triplet pattern of (Gly-Pro-Y) or (Gly-X-Hyp) in each chain. The specific order of these amino acids forms the foundational structure of collagen. One of the most prevalent triplets is Pro-Hyp-Gly, accounting for approximately 10% of the total amino acid composition^29^^,^^31^. The secondary structure of collagen is characterised by a left-handed helical conformation, specifically of the polyproline-II type. This structure can coil or fold along defined axes to produce various spatial configurations, including α-helices, β-sheets, β-turns and irregular arrangements^43^. These configurations are subsequently organised into triple-helical structures that exhibit a right-handed superhelical arrangement^44^. In the triple-helix structure, each polypeptide chain comprises over 1000 amino acids. Among these amino acid sequences, there is a repeating and alternating tripeptide motif, designated as GXY, where the third amino acid must be glycine. Glycine, having a smaller side chain, is better suited for positioning within the interior of the triple helix, thereby facilitating the formation of the triple helix. This arrangement results in the alpha chains being more tightly packed along the central axis of the trimeric molecule^45^. X and Y positions are typically occupied by proline and lysine, respectively. During translation, these amino acids undergo hydroxylation through enzymatic modification, resulting in the production of hydroxyproline and hydroxylysine. These modified residues can form interchain bonds with glycine, thereby enhancing the stability of the triple-helix structure. Furthermore, the side chains of proline and hydroxyproline possess a cyclic structure, which imposes a ring restriction between the α-carbon and the amide nitrogen atom. As a result, the side chains of proline and hydroxyproline cannot rotate freely, contributing to the stability of the left-handed α-helical structure and facilitating the formation of the collagen triple helix^46^.

The traditional perspective posits that the *C*-terminal prepeptide structural domain is crucial for the assembly of both homo- and heterotrimers. In the formation of heterotrimers, the *C*-terminal G2 structural domain of the R2 chain interacts with the G2 structural domain of the R1 chain within the *C*-terminal prepeptide structural domain, resulting in the formation of a stabilised dimer. This dimerisation subsequently leads to the completion of the trimer assembly^47^. In a recent study, Yammine et al. demonstrated that the *C*-terminal amino acid sequences of both the *C*-terminal prepeptide domain and the triple-helix domain collaboratively regulate the assembly of type I collagen trimers. The α1 chain triple-helix domains are rich in proline and hydroxyproline residues at the *C*-terminal end, which significantly enhances the stability of the triple-helix structure, allowing for rapid and irreversible folding into triple helices. In contrast, the *C*-terminus of the α2 chain’s triple-helix structural domain exhibits lower stability, which prevents it from rapidly adopting a triple-helix conformation. This synergistic interaction between the *C*-terminal prepeptide domain and the triple-helical domain is essential for the successful formation of the collagen triple helix^48^. Meanwhile, MacKerell et al. employed molecular simulations to demonstrate that the folding of collagen molecules occurs through a step-by-step process. Initially, two chains form a transient template, followed by the folding of a third chain onto this template, thereby completing a cycle of fold propagation^49^. The formation of both heterotrimers and homotrimers of collagen is intricately linked to gene regulation and structural functionality. Collagen α-chain genes can modulate their tissue-specific expression. For instance, fibroblasts co-express the COL1A1 and COL1A2 genes, resulting in the assembly of heterotrimeric type I collagen, which is crucial for the strength of skin and bone. In contrast, chondrocytes exclusively express the COL2A1 gene, leading to the production of homotrimeric type II collagen. Structurally, the formation of triple helices necessitates precise alignment of the Gly-X-Y repeat sequence. Homotrimers achieve symmetry through identical sequences, while heterotrimers depend on complementary sequences and chaperone proteins to stabilise their folding. Functionally, heterotrimers expand the roles of collagens: type IV collagens contribute to the formation of basement membrane networks through non-protofibrillar interactions that are facilitated by chain-specific binding domains of laminin and proteoglycans. Conversely, homotrimers, such as type II collagen, promote a homogeneous protofibrillar organisation in cartilage. Heterotrimers provide diverse mechanical properties and interactions with extracellular matrix components, whereas homotrimers optimise tissue-specific functions, including resistance to cartilage compression. The evolutionary conservation of homotrimers, paired with the diversification of heterotrimers, balances structural stability and functional adaptation, particularly in response to diseases such as osteogenesis imperfecta, where mutations interfere with trimer assembly. Thus, the diversity of collagen trimers underscores their evolutionary adaptation to meet the specific requirements of different tissues^44^^,^^50^^,^^51^.

The tertiary structure of collagen refers to its overall three-dimensional configuration, which builds upon the secondary structure. In this stage, the peptide chains are further coiled and folded into a specific spatial arrangement stabilised by secondary bonds. The amino acid chains arrange themselves into a triple-helical structure, simultaneously, intra- and intermolecular cross-linking processes, such as alcohol-formaldehyde condensation, contributing to the stability of the collagen molecule^52^. The quaternary structure of collagen involves the assembly of peptide chains derived from the tertiary structure, resulting in the formation of collagen complexes with distinct functional properties and stable triple-helical configurations. The cross-linking of peptide chains within the molecule leads to the formation of larger structural units, which further enhances the stability of collagen, providing greater tensile strength and resistance^21^^,^^53^. Therefore, collagen possesses three distinctive structural features: (1) the amino acid repeat sequence [Gly-X-Y]n remains uninterrupted; (2) hydroxyproline and proline occupy the X and Y positions, respectively; and (3) three left-handed polyproline α-strands of equal length coalesce to form a right-handed triple helix. These characteristics contribute to collagen’s unique four-level structure^54^.

### Biosynthesis of collagen

Collagen synthesis begins in the nucleus of fibroblasts located within the skin. Within the nucleus, specific collagen genes are transcribed into pro-α1 and pro-α2 messenger RNAs (mRNAs) ^55^. These mRNAs transport genetic information encoding the collagen polypeptide chain from the nucleus to the cytoplasm, where they bind to ribosomes. Within the ribosomes, the mRNAs are translated into specific amino acid sequences that form pre-collagen polypeptide chains. Subsequently, these polypeptide chains are transported to the endoplasmic reticulum, where they undergo modifications. Among these intracellular modifications are the hydroxylation of specific lysine and proline residues. This process leads to the formation of hydroxylysine, 3-hydroxyproline and 4-hydroxyproline. Additionally, certain hydroxylysine residues undergo glycosylation to yield galactosylhydroxylysine and glucosylhydroxylysine^42^^,^^56^^,^^57^. This step is essential for stabilising the collagen triple-helix structure. The modified polypeptide chain subsequently folds to form a stable triple-helix structure. This structure consists of three polypeptide chains intricately entwined, specifically two pro-α1 chains and one pro-α2 chain, which are bound to each other near the *C*-terminus. The triple-helical structure extends towards the *N*-terminus in a zipper-like manner, resulting in the formation of a pre-collagen molecule^58^. The procollagen molecule is subsequently translocated to the Golgi apparatus, where it is subjected to final modifications before the secretion of its triple-helical structure into the EMC via vesicular transport. Extracellularly, procollagen undergoes proteolytic cleavage by collagen peptidases, resulting in the removal of the non-helical regions at both the *N*- and *C*-terminus^59^. This process ultimately yields tropocollagen. The formation of covalent bonds between tropocollagen molecules facilitates their spontaneous assembly into collagen fibres. Additionally, a non-helical region exists at the terminus of the collagen molecule, where lysine and hydroxylysine residues are modified to form aldehyde groups through the action of lysyl oxidase. These aldehyde groups can then condense with lysine residues on neighbouring collagen molecules, resulting in the formation of cross-linking bonds. This process enhances the structural stability of the collagen fibres^60^^,^^61^.

## Mechanisms and pathways promoting collagen synthesis

### Pathways associated with TGF-β/Smad

#### TGF-β/Smad pathway

TGF-β is a functional protein that is expressed by all cells in the human body. It plays a critical role in maintaining normal development and homeostasis, regulating various cellular activities such as cell proliferation and differentiation^62^. In fibroblasts, TGF-β serves as the most potent stimulator of collagen production and can activate the expression of the ECM genes, including those for type I collagen.

The TGF-β superfamily comprises nearly 30 proteins in mammals, including activins, junctional proteins, bone morphogenetic proteins (BMPs), TGF-βs, growth and differentiation factors (GDFs) and macrophage migration inhibitory factor (MIF). Within this superfamily, there are three isoforms of TGF-β, including TGF-β1, TGF-β2 and TGF-β3^63^^,^^64^.

**Figure 1.**
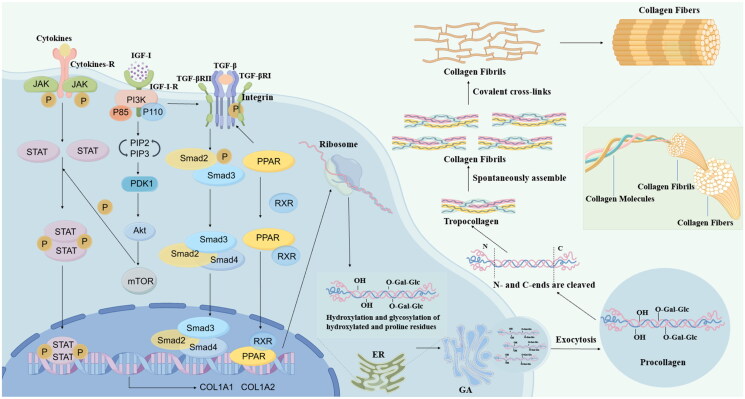
The biosynthesis process of collagen and related pathways. (This figure was drawn by the authors by Figdraw, www.figdraw.com).

Activin receptor-like kinase 5 (ALK5) and TβRII are essential for mediating TGF-β signalling; both are enzyme-linked receptors exhibiting dual functions as serine/threonine kinases and tyrosine kinases. The intracellular signalling pathways of the TGF-β family can be categorised into typical and atypical pathways. The typical TGF-β signalling pathway, commonly referred to as Smad signalling, involves Smad family proteins. These Smad proteins regulate numerous genes that encode components of the ECM, including collagen, laminin, fibronectin and proteoglycans. TGF-β initially binds to either TGF-β receptor III or TβRII, subsequently recruiting TβRI to form the TGF-β-TβRI-TβRII complex. This complex activates the type I TGF-β receptor through phosphorylation, initiating intracellular signalling. Following this activation, the *C*-terminal SXS motifs in Smad2 and Smad3 are phosphorylated and subsequently dissociate from the receptor. Smad2 and Smad3 subsequently associate with Smad4 to form a complex that translocates to the nucleus, where it modulates the transcription of the COL1A1 and COL1A2 genes^65–70^ (Figure 1). The α-chain serves as the fundamental unit of collagen, with the COL1A1 and COL1A2 genes encoding the pre-α1 and pre-α2 chains of type I collagen, respectively^71^. This process promotes collagen transcription. Additionally, connective tissue growth factor (CTGF), a cysteine-rich peptide synthesised and secreted by fibroblasts, acts as a downstream mediator of TGF-β-induced fibroblast proliferation, effectively inducing collagen synthesis^72^. In muscle cells, TGF-β promotes the expression of type I collagen by stimulating the production of CTGF and fibroblast growth factor 2 (FGF-2), which further increases type I collagen expression^69^. Fan et al. utilised a 5-week-old female BALB/c nude mouse model to demonstrate that hyaluronic acid cross-linked fillers could stimulate the synthesis of type I collagen and elastin fibres in the skin by activating the TGF-β pathway^73^. Liu et al. employed a skin fibroblast model to demonstrate that collagen peptides could enhance collagen synthesis and impede collagen degradation by activating the TGF-β. This mechanism contributes to the repair of photoaged skin cells^74^. Using normal human dermal cells and a hairless mouse model, Hwang et al. verified that clove extract could promote collagen synthesis by activating the TGF-β/Smad pathway by detecting the phosphorylation of Smad2/3 and the level of Smad7^75^.

In summary, the upregulation of TGF-β expression enhances collagen synthesis, while the activation of this pathway stimulates the production of EMC proteins. These findings suggest that compounds capable of activating the TGF-β pathway may promote collagen synthesis and have the potential to delay skin ageing.

#### PI3K/Akt pathway

The activation of the PI3K/Akt pathway not only enhances the expression of vascular endothelial growth factor (VEGF) but also facilitates cell proliferation, migration, angiogenesis and collagen synthesis, collectively stimulating the wound-healing process^76–79^.

PI3K is a vital cellular signalling protein that specifically catalyses the phosphorylation of the hydroxyl group at the third position of phosphatidylinositol. It is classified into three distinct classes: I is a heterodimeric lipid kinase composed of a structural catalytic domain p110 and a regulatory domain p85. This class is capable of activation by various receptors, including G protein-coupled receptors, insulin-like growth factor I (IGF-I), receptor tyrosine kinases (RTKs) and B-cell receptors, thereby regulating cell proliferation, growth and metabolism. II consists of a single p110 catalytic subunit. III includes vacuolar protein sorting 34 (Vps34)^80^. These classes illustrate the diverse roles and regulatory mechanisms of PI3K in cellular processes.

When growth factors bind to RTKs or G protein-coupled receptors on the cell membrane, they activate class Ia and Ib PI3K isoenzymes, respectively. This activation stimulates PI3K to phosphorylate phosphatidylinositol 4,5-bisphosphate (PIP2), producing phosphatidylinositol 3,4,5-trisphosphate (PIP3). The generation of PIP3 subsequently activates 3-phosphoinositide-dependent protein kinase-1 (PDK1), which phosphorylates the threonine 308 site of Akt. Akt then forms a complex with the lipid products, leading to its further phosphorylation at the serine 473 site by PDK2 and complete activation. Once activated, Akt regulates numerous cellular functions by interacting with various relevant molecules, including antiproliferative molecules P21, P27, TGF-β and gamma-aminobutyric acid (GABA) receptors^76^^,^^81–84^ (Figure 1). Hubchak et al. investigated the effects of Rac1 on human mesangial cells (HMC) model and found that Rac1 significantly enhanced type I collagen synthesis induced by TGF-β. This enhancement occurred through the activation of the PI3K signalling pathway^85^. Yokoyama et al. demonstrated that PI3K/Akt pathway activates the TGF-β signalling pathway, thereby inducing type I collagen synthesis in a retinal pigment epithelium (RPE) model^23^. The PI3K/Akt pathway is recognised to be associated with collagen synthesis. Inhibition of this pathway results in the downregulation of collagen expression, whereas its activation enhances collagen synthesis.

In conclusion, compounds that activate the PI3K/Akt signalling pathway may promote collagen synthesis, potentially delaying skin ageing. IGF-I, which is essential for cell growth, differentiation, survival, protein synthesis, motility and proliferation, has become a promising therapeutic target for combating skin ageing^86^. As an upstream regulator of the PI3K/Akt pathway, the further enhancement of IGF-I expression, plays a significant role in the anti-ageing processes of the skin.

#### PPARβ/δ pathway

Belonging to the nuclear hormone receptor superfamily, PPARs are transcription factors that are activated by specific ligands. These factors play a crucial role in regulating various cellular metabolic processes, such as differentiation and proliferation of cells, maintenance of lipid homeostasis and energy metabolism^87^.

PPARs are primarily classified into three classifications: PPARα, PPARβ/δ and PPARγ^88^. PPARβ/δ, in particular, is structured with four functional domains: A/B, C, D and E/F. The A/B domain contains the *N*-terminal activation function-1 (AF-1) domain, which is crucial for ligand binding and activation of PPARs. The C domain serves as the DNA-binding domain (DBD), featuring two zinc finger motifs and eight conserved cysteine residues. This region is responsible for recognising gene promoters, enabling nuclear receptors to bind to target genes in various ways. The D hinge region links the DBD to the ligand-binding domain (LBD). Multiple cofactors interact with PPARs through the D region, which is essential for their transcriptional activity. Notably, the D hinge, A/B and E regions are less conserved compared to other regions. The E/F region comprises the LBD, a dimerisation interface and the ligand-dependent activation domain AF-2, which specifically binds endogenous or exogenous lipophilic ligands. Ligand binding stabilises the E/F region structurally and induces a conformational alteration that facilitates interaction with the coactivation complex. Within this context, the AF-2 domain is particularly important for recruiting PPAR cofactors, playing a critical role in the transcriptional process^89–92^. The retinoid X receptor (RXR) recognises specific nucleotide sequences known as PPAR response elements (PPREs), which are situated in the regulatory regions of its target genes^93^ (Figure 1). A direct repeat (DR1 or DR2) of the hexameric nucleotide sequence AGGTCA, separated by one or two nucleotides, is featured in the promoter regions of genes targeted by PPAR^94^. PPAR-RXR complexes bind to these sequences to activate the expression of target genes. Research has demonstrated that TGF-β is a molecular target of the PPAR. Specifically, PPARδ can induce the expression of TGF-β, a downstream signalling molecule, by attaching to the DR1-type PPRE situated in the promoter region of the TGF-β gene, thereby promoting collagen synthesis^93^. Using skin fibroblasts and a male mouse model, Ham et al. investigated the expression of type I collagen and confirmed that PPARδ promotes collagen synthesis through the activation of the TGF-β/Smad signalling pathway^87^. Jung et al. assessed the effects of magnesium salts of salvianolic acid B on collagen synthesis in an aged rat model. They found that these magnesium salts significantly increased TGF-β expression through the activation of PPARδ, thereby promoting the synthesis of type I collagen^95^. Kwok et al. conducted a study utilising a fibroblast cell model, which revealed that treatment with ginsenoside Rb1 significantly enhanced the expression levels of type I collagen. The experimental findings also confirmed that ginsenoside Rb1 regulates type I collagen expression through the PPAR pathway^96^.

Given these studies, PPARβ/δ is anticipated to be a potential target for anti-skin ageing therapies. We hypothesise that targeting and activating PPARβ/δ could further stimulate the TGF-β/Smad pathway, thereby enhancing collagen synthesis for skin anti-ageing applications.

#### JAK/STAT pathway

The JAK2/STAT3 pathway is crucial for collagen synthesis as well as for cellular processes such as proliferation, differentiation and migration^97^. The JAK family comprises JAK1, JAK2, JAK3 and Tyk2, which serve as essential downstream signalling components of receptor kinases on the cell membrane, playing a pivotal role in signal transduction. The signal transducer and activator of the transcription (STAT) family include STAT1, STAT2, STAT3, STAT4, STAT5a, STAT5b and STAT6^98^. Each STAT protein possesses a distinct biological function and plays a crucial regulatory role in cell differentiation, metabolism and immune responses. Structurally, STAT proteins possess several key domains: coiled-coil domain (CCD), *N*-terminal domain (ND), DBD, linker domain (LD), transcription activation domain (TAD) and SH2 domain. Upon the binding of a cytokine to its corresponding transmembrane receptor, signalling to the intracellular domain initiates the tyrosine phosphorylation and activation of the associated JAKs. This action subsequently leads to the phosphorylation of STAT proteins on the receptor’s tyrosine residues, facilitating the dissociation of STAT from the receptor. The phosphorylated STAT then interacts with the receptor through the SH2 domain-phosphotyrosine interaction, resulting in the formation of either homodimers or heterodimers. These dimers are subsequently translocated from the cytoplasm to the nucleus, where they bind to target gene promoters to regulate transcription^98–100^ (Figure 1). Research has demonstrated that the TGF-β pathway can activate the JAK2/STAT3 signalling pathway, thereby enhancing collagen synthesis. JAK2, a non-receptor tyrosine kinase, is phosphorylated and activated in response to TGF-β1 stimulation. Once activated, JAK2 further phosphorylates the STAT3 protein, leading to its dimerisation and translocation to the nucleus. Within the nucleus, STAT3 binds to specific DNA sequences to regulate the expression of collagen and other genes associated with fibrosis^101^. Additionally, STAT3 directly interacts with the upstream promoter regions of collagen genes, promoting the expression of type I and type III collagen^102^. Furthermore, STAT3 also plays a critical role in regulating other genes related to collagen synthesis, including CTGF and α-smooth muscle actin (α-SMA)^103^^,^^104^. Using a hepatic stellate cell (HSC) model, Gu et al. demonstrated that the JAK inhibitor SHR0302 may reduce the synthesis of type I and type III collagen by inhibiting the JAK/STAT3 signalling pathway^97^. In contrast, Niu et al. investigated a model of HSCs treated with leptin and found that leptin promotes type I collagen synthesis through the activation of the JAK pathway^105^.

In summary, the JAK2/STAT3 signalling pathway is crucial for collagen synthesis and is closely associated with the progression of various fibrotic diseases. Consequently, this pathway presents a potential therapeutic target, as regulating its activity may help modulate collagen synthesis and contribute to anti-ageing effects in the skin.

### Integrin-related pathways

Integrins are cell surface receptors that facilitate the connection between the ECM and the intracellular actin cytoskeleton. They interact with various proteins in the ECM, notably collagen. This interaction is crucial for processes such as cell adhesion, migration, proliferation and signalling^106–109^.

Integrins in mammals are large transmembrane heterodimers formed by the noncovalent binding of α and β subunits^110^. Each subunit consists of extracellular domain, transmembrane (TM) domain and cytoplasmic tail (CT). The CT of integrins plays a crucial role in both their activation and signalling. The extracellular domain of the α subunit comprises the I domain, the β-propeller domain, the thigh domain and two calf domains (Calf-1 and Calf-2). It also features a metal ion-dependent adhesion site that binds divalent cations, which is essential for ligand binding. Additionally, the cytoplasmic regions of both subunits contain a highly conserved GFFKR sequence near the membrane, which regulates integrin activity. In contrast, the extracellular region of the β subunit includes an I-like domain, a hybrid domain, a plexin-semaphorin-integrin (PSI) domain, four cysteine-rich epidermal growth factor (EGF) domains and a β-tail domain^111^^,^^112^. Integrins can be classified into four subfamilies based on the specificity of the ligands they recognise: collagen-binding, laminin-binding, arginine-glycine-aspartate-binding and leukocyte-specific integrins^113^. Collagen primarily interacts directly with a specific subset of four β1 integrins: α1β1, α2β1, α10β1 and α11β1^114^. Among these, integrin α2β1 serves as the principal receptor for type I collagen, specifically recognising the aspartate-glycine-glutamate-alanine (DGEA) sequence in the α1 chain of type I collagen. This interaction regulates the transcription of collagen genes and contributes to the formation of a stable triple-helical structure of collagen^107^^,^^115^. The adhesion of integrins to target cells activates various intracellular signalling pathways, including integrin downstream signalling, the TGF-β pathway and the JAK/STAT pathway^116^^,^^117^. Integrins can activate the TGF-β pathway by binding to the structural domains of the TGF-β precursor, which leads to its activation and the subsequent release of the mature TGF-β growth factor^106^. These pathways collectively influence collagen behaviour and gene expression (Figure 1).

### Proline hydroxylase and lysine hydroxylase-related pathways

Lysine hydroxylase and proline hydroxylase play important roles in collagen synthesis^118^^,^^119^ (Figure 1). Within the endoplasmic reticulum, these enzymes catalyse the hydroxylation of lysine and proline residues in the peptide chains of collagen precursors^61^. Hydroxylation is a critical step in the formation of the collagen triple helix, as it enhances hydrogen bonding between the peptide chains, thereby improving the stability of the triple-helix structure. Specifically, proline hydroxylase catalyses the hydroxylation of proline. Insufficient hydroxylation of procollagen results in an inability to form a stable triple-helical structure, which prevents its excretion from the cell. This condition adversely affects the rate of collagen synthesis and disrupts normal functional processes. Lysine hydroxylase is responsible for catalysing the hydroxylation of lysine, leading to the formation of hydroxylysine. This modification is crucial for creating cross-links both within and between collagen molecules, significantly enhancing the mechanical strength of collagen fibres^120^.

The hydroxylation reaction is the rate-limiting step in collagen synthesis^121^^,^^122^. This reaction not only enhances the stability of collagen but also facilitates the secretion of procollagen. When collagen remains unhydroxylated, it cannot properly fold to form a functional fibre structure, adversely affecting its role in tissues^58^. Consequently, proline hydroxylase and lysine hydroxylase are essential for effective collagen synthesis. In summary, by stimulating the activity of proline hydroxylase and lysine hydroxylase, collagen synthesis within the organism can be enhanced, leading to anti-ageing effects on the skin.

## Natural products and small molecule compounds with collagen synthesis-promoting effects

### Natural products with collagen synthesis-promoting effects

#### Esculetin

**Figure 2.**
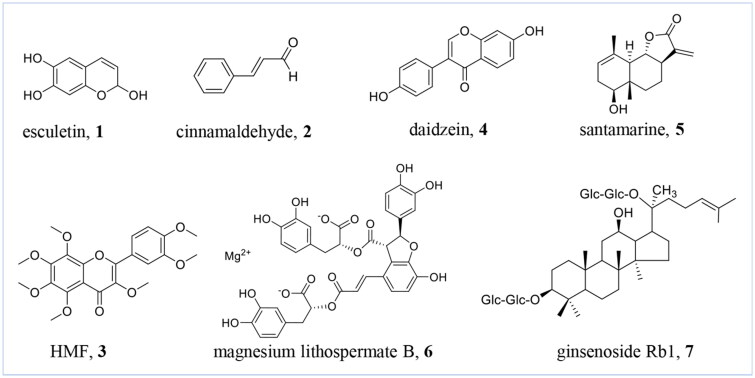
Chemical structures of natural products with collagen synthesis promoting effects **1-7**. (This figure was drawn by the authors).

Esculetin (6,7-dihydroxycoumarin) (**1**) (Figure 2) is a coumarin-like natural product obtained from various sources, including the bark of the trunk and the skin of the twigs from the Chinese herbal medicine, *Fraxinus rhynchophylla* Hance. This natural product exhibits an array of beneficial activities, such as anti-inflammatory, anticancer, hypoglycaemic, neuroprotective, cardiovascular protective and antibacterial effects^123^. Park et al. investigated its effect on type I procollagen expression in human dermal fibroblasts (HDFs) and found that esculetin significantly increased both the protein and mRNA levels of type I procollagen within concentrations range of 0.1 to 100 μM, exhibiting a dose- and time-dependent effect. Additionally, esculetin treatment resulted in significant increases in the phosphorylation levels of ERK1/2, p38, JNK and Akt, as well as upregulated expression of the Sp1 protein. More importantly, Sp1 may play a crucial role in mediating esculetin-induced expression of type I procollagen^124^.

#### Cinnamaldehyde

Cinnamaldehyde (**2**) (Figure 2), predominantly found in the volatile oil of cinnamon bark and leaves, possesses a variety of biological functions, such as antifungal, antioxidants, anti-inflammatory, hypoglycaemic and anti-cancer activities^125^^,^^126^. In experiments conducted by Takasao et al., under the influence of cinnamaldehyde concentrations ranging from 5 to 20 μM, enhanced the levels of pro-type I collagen in HDFs in a dose-dependent manner. Additionally, at a concentration of 10 μM, compound **2** markedly induced the phosphorylation of IGF-I receptors. The impact of cinnamon extract on the mRNA expression of the type I collagen α1 subunit was assessed using real-time polymerase chain reaction (RT-PCR), revealing that cinnamaldehyde increased the mRNA expression of type I collagen in NB1RGB cells by approximately 1.6-fold. In summary, cinnamaldehyde enhances both the mRNA and protein expression levels of type I collagen by activating the IGF-I receptor target, indicating its potential to promote collagen synthesis. Importantly, it demonstrated minimal toxicity to cells, suggesting its promise as a therapeutic agent for combating skin ageing^127^.

#### Flavonoid derivatives

##### HMF

HMF (3,5,6,7,8,3′,4′-heptamethoxyflavone) (**3**) (Figure 2) is a flavonoid natural product derived from citrus fruits, noted for its neuroprotective, anti-inflammatory, anti-ageing and photoprotective properties^128^^,^^129^. Kim et al. observed that treatment with HMF at concentrations of 50 μg/mL, 100 μg/mL and 200 μg/mL led to a dose-dependent enhancement of the type I procollagen protein content in HDFs, with levels rising from approximately 0.4 μg/mL in the control group to 0.6 μg/mL, 0.8 μg/mL and 1.8 μg/mL, respectively. Additionally, HMF inhibited MMP expression in UVB-induced HDFs while significantly enhancing the expression of type I procollagen, reaching levels approximately 4.5 times exceeding those of the control group at a concentration of 200 μg/mL concentration. Furthermore, HMF increased the expression of Smad3 protein in UVB-induced HDFs and significantly decreased the expression of Smad7 protein at both 100 μg/mL and 200 μg/mL concentrations. In conclusion, HMF upregulates the expression of type I procollagen through modulation of the TGF-β signalling target. The underlying mechanism is likely related to the upregulation of Smad3 protein expression accompanied by the downregulation of Smad7 protein expression, which collectively promotes the activation of the TGF-β receptor target and enhances the synthesis of type I procollagen^129^.

##### Daidzein

Daidzein (**4**) (Figure 2), also known as 7-hydroxy-3–(4-hydroxyphenyl)-4H-chroman-4-one, is classified among isoflavone derivatives. It is present in various plants, including red clover, soybeans, alfalfa and other legumes. Daidzein typically exists in the form of glycosides and has been shown to possess therapeutic effects against breast cancer, cardiovascular disease and osteoporosis^130^^,^^131^. Zhao et al. identified that daidzein at concentrations of 5 μg/mL and 50 μg/mL significantly activated the transcription of type I collagen in a luciferase assay. Notably, the luciferase activity in the 50 μg/mL treatment group was nearly three times higher than that of the control group. Furthermore, RT-PCR analysis indicated that daidzein significantly upregulated type I collagen mRNA expression, achieving a 38% increase at the 50 μg/mL concentration. Furthermore, it significantly decreased the expression of MMPs and activated the phosphorylation of Smad2/3. Moreover, immunohistochemical analysis revealed a significant increase in the positive staining of type I collagen in the skin following treatment with daidzein^132^.

In brief, daidzein promotes the synthesis of type I collagen and inhibits its degradation in the skin by activating the TGF-β/Smad. This action results in increased collagen accumulation, as demonstrated in both *in vivo* and *in vitro* experiments.

#### Santamarine

Santamarine (**5**) (Figure 2), a sesquiterpene lactone derived from *Artemisia annua* of the Asteraceae family, demonstrates a variety of biological functions including antimicrobial, anti-inflammatory and anticancer effects^133^. Oh et al. investigated the impact of santamarine on photoageing using a UVA-induced model of HDFs. Their findings revealed that santamarine possesses dose-dependent free radical scavenging activity and significantly enhances the expression of type I procollagen. In UVA-irradiated HDFs, santamarine markedly increased the protein levels of TGF-β and Smad2/3. In conclusion, santamarine promotes procollagen production by inducing TGF-β, while mitigating the UVA-induced upregulation of MMP expression^134^.

#### Magnesium lithospermate B

Magnesium lithospermate B (**6**) (Figure 2) is a water-soluble phenolic acid primarily extracted from the roots and rhizomes of *Salvia miltiorrhiza*. It is typically found in various salt forms and is recognised for its anti-inflammatory, anti-oxidation and antifibrotic activities^135^^,^^136^. Research by Jung et al. demonstrated that magnesium lithospermate B binds to the active sites of the PPAR β/δ receptor, enhancing its DNA-binding activity. Notably, at a concentration of 10 μM, magnesium lithospermate B exhibited more potent activation of PPARβ/δ compared to the established agonist GW501516 and promoted increased nuclear translocation of PPARβ/δ in HDFs. Subsequent studies utilised an aged Sprague-Dawley rat skin model to further explore the effects of magnesium lithospermate B on PPARβ/δ activity and the expression of ECM proteins. The results revealed that PPARβ/δ nuclear levels were lower in the control group compared to younger rats, while a dose-dependent increase in PPARβ/δ levels was observed in the magnesium lithospermate B-treated group. Furthermore, treatment with magnesium lithospermate B significantly elevated the protein levels of TGF-β1, COL1A1 and COL3A1 when compared to the untreated group. Additionally, UVB irradiation led to a decrease in PPARβ/δ nuclear levels, but magnesium lithospermate B effectively prevented this decline in a dose-dependent manner. UVB exposure also reduced the protein levels of TGF-β1, COL1A1 and COL3A1, whereas magnesium lithospermate B restored their expression. In summary, magnesium lithospermate B markedly upregulates the expression of TGF-β1 and collagen types I and III through the activation of PPARβ/δ, suggesting its potential role in counteracting both natural and UVB-induced skin ageing^95^.

#### Ginsenoside Rb1

Ginsenoside Rb1 (**7**) (Figure 2) is a dammarane triterpenoid saponin compound extracted from ginseng root. It offers a range of beneficial effects, including cardiovascular and central nervous system activities, as well as antidiabetic and antitumor properties^137^^,^^138^. Kwok et al. found that ginsenoside Rb1 increased the expression of type I collagen in HDFs in a manner that was dependent on both dose and duration, across a concentration range of 0.1 to 50 μM. Treatment of HDFs with 30 μM ginsenoside Rb1 for 24 h resulted in a 1.8-fold increase in the mRNA expression of COL1A1. Additionally, luciferase reporter gene assays demonstrated that ginsenoside Rb1 activated the transcriptional activity of PPARδ at concentrations of 10 to 100 μM. The critical role of PPARδ in Rb1-induced type I collagen expression was further validated using PPARδ-specific antagonists and siRNA assays. Furthermore, miR-25, identified as a negative regulator of COL1A2 protein expression, directly inhibits type I collagen expression. However, ginsenoside Rb1 mitigated this inhibitory effect by reducing miR-25 levels, thereby promoting type I collagen expression. To summarise, ginsenoside Rb1 upregulates the expression levels of both mRNA and protein for type I collagen by activating the PPARδ signalling pathway, thereby promoting collagen synthesis. Additionally, ginsenoside Rb1 exhibits no cytotoxicity towards cells, highlighting its potential as an effective therapeutic agent for combating skin ageing^96^.

### Potentially small molecule compounds with collagen synthesis-promoting effects

This review discusses several pathway targets relevant to collagen synthesis, including the TGF-β/Smad the JAK/STAT, the PPARδ, the PI3K/Akt, the integrin-associated pathway and the rate-limiting enzymes involved in collagen synthesis. While the activation of these pathways is known to promote collagen synthesis, there have been no reports of small molecule compounds that directly activate these pathways, or enhance lysine and proline hydroxylase activity.

It has been shown that activating the PPARδ pathway can more effectively promote collagen synthesis. Therefore, this review focuses on several small molecule compounds that exhibit potent agonistic activity towards PPARδ. Although these agonists are not currently employed in anti-skin ageing therapies, they possess the potential to activate the PPARδ pathway and significantly enhance collagen synthesis, thus providing a promising strategy for combating skin ageing.

#### [1,2,4] Thiadiazole PPARδ agonists

**Figure 3.**
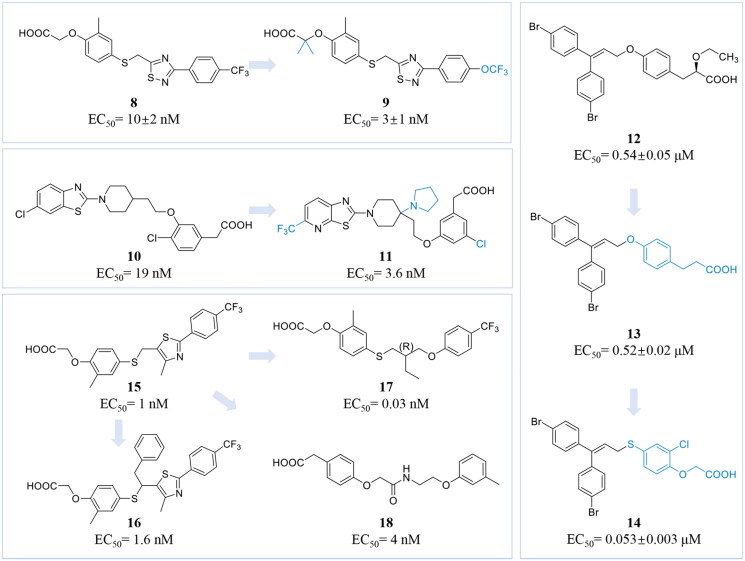
Chemical structures of representative PPARδ agonists **8-18**. (This figure was drawn by the authors).

Shen et al. identified a selective PPARδ agonist that demonstrated significant *in vivo* efficacy and a favourable safety profile. Methylthiazole derivatives were selected as lead compounds for structure-activity relationship studies. Through the screening of various five- and six-membered heteroaryl and heterocyclic groups, the compound resulting from the substitution of the methylthiazole at the 5-position with [1,2,4] thiadiazole was ultimately identified as compound **8** (Figure 3). It exerted submicromolar activity against PPARα (EC_50_ = 468 ± 10 nM) and showed potent activity against PPARδ (EC_50_ = 10 ± 2 nM), while demonstrating no activity against PPARγ (EC_50_ > 3000 nM). Additionally, the introduction of additional bis-methyl groups in the [1,2,4] thiadiazole series was found to enhance its’ activity against both PPARδ and PPARα. Further modification, where CF_3_ was replaced with OCF_3_, obtained compound **9** (Figure 3), which displayed approximately twice the potency of the CF_3_ analogue (compound **8**) against PPARα (EC_50_ = 33 ± 1 nM) and PPARδ (EC_50_ = 3 ± 1 nM), although its activity against PPARγ remained unchanged (EC_50_ > 3000 nM). In summary, compound **9** exhibits efficient and highly selective dual agonism for PPARα and PPARδ^139^.

#### 4-(1-Pyrrolidinyl) piperidine PPARδ agonists

Kato et al. identified a series of piperidinyl and piperazinyl benzothiazole-based PPARδ agonists through virtual screening. Initial molecular docking studies of the lead compound **10** (EC_50_ = 19 nM) (Figure 3) with human PPARs demonstrated that the deployment of a bulky hydrophobic group into the central piperidine ring enhanced its binding activity to PPARδ while increasing selectivity. It was found that a substituent on the benzene ring at the 3-position exhibited increased activity. However, the modified compound also inhibited cytochrome P4502C9 due to excessive hydrophobicity, resulting in metabolic instability in human liver microsomes. Subsequent substitutions of carbon atoms adjacent to the piperidine ring with nitrogen revealed that the pyrrolidinyl analogue exhibited the best activity. Additionally, replacing the chlorine atom in the benzothiazole moiety with a trifluoromethyl (CF_3_) group improved both activity and stability, although this modification was associated with the occurrence of cardiac arrhythmias. The research culminated in modifying the left side of the molecule by incorporating a nitrogen atom into the benzothiazole ring, providing compound **11** (Figure 3), which exhibited an EC_50_ of 3.6 nM and demonstrated low hydrophobicity (CLog*P* = 3.0). In summary, compound **11** displayed high activity and selectivity for PPARδ^140^.

#### Bis (4-bromophenyl) methane PPARδ agonists

Sauerberg et al. identified a structurally novel PPARδ agonist, compound **12** (EC_50_ = 0.54 ± 0.05 μM) (Figure 3), from a high-throughput screening of approximately 100 compounds utilising a cell-based transactivation assay. This compound is a non-selective PPAR agonist, exhibiting moderate activity against all three PPAR isoforms. Subsequent structural optimisation involved the removal of the ethoxy group while maintaining the hydrophobic portion, resulting in the development of the PPARδ-selective agonist, compound **13** (EC_50_ = 0.52 ± 0.02 μM) (Figure 3). However, compound **13** did not demonstrate an allosteric effect comparable to the reference compound in the *in vitro* free fatty acid oxidation assay. Pharmacokinetic (PK) evaluations of compound **13** in rats indicated poor oral bioavailability and a short half-life. By comparing published crystal structures of PPARδ receptor proteins, the research team modified the central benzene ring and synthesised a series of compounds. Among these, compound **14** (EC_50_ = 0.053 ± 0.003 μM) (Figure 3) displayed favourable pharmacokinetic properties, including good oral bioavailability and a sufficiently long half-life^141^.

#### GW501516 derivatives

GW501516 (**15**) (EC_50_ = 1 nM) is the first selective agonist of PPARδ to undergo clinical trials. Its structure features a head consisting of a carboxylic acid group and a benzene ring, a connecting chain composed of a heterocyclic ring and a flexible carbon chain and a tail composed of a heteroaromatic or aromatic hydrophobic group. This configuration facilitates the formation of hydrogen bonds with the AF-2 region of PPARδ, thereby enhancing its binding affinity to the PPARδ receptor. However, GW501516 was found to rapidly induce cancer in late-stage animal studies, leading to the discontinuation of its clinical trials^142^. Currently, GW501516 serves as a lead compound in the search for novel, selective and potent PPARδ agonists.

Ham et al. structurally modified GW501516 based on molecular docking experiments, leading to the discovery of a sequence of Y-shaped PPARδ agonists. This was accomplished by substituting α-position of the sulphide. It was found that the activity decreased linearly with the increasing length of the substituent alkyl side chain. Among the derivatives, the incorporation of a short phenyl-alkyl group resulted in compound **16** (EC_50_ = 1.6 nM) (Figure 3), exhibiting no activity against PPARα and PPARγ at concentrations of up to 10 μM. This demonstrated that compound **16** possesses potent and selective agonist activity for PPARδ^143^.

Zhang et al. identified a range of novel Y-shaped alkyl thiophenoxyacetic acids by modifying the structure of compound **15**. Most of the synthesised compounds exhibited significant PPARδ-specific agonistic activity. The study indicated that the chirality of the Y-intersection, along with the lengths of the backbone and side arms, is crucial for agonist activity at PPARδ. This study demonstrated that both the chirality of the Y-intersection and the length of the side arms and backbone are crucial for the agonist activity at PPARδ. Among them, compound **17** (EC_50_ = 0.03 nM) (Figure 3) exhibited potent activity along with advantageous pharmacokinetic characteristics^144^. Da’adoosh et al. discovered new hits and leads using structure-based computational tools. Their approach integrated two distinct analytical techniques: iterative stochastic elimination (ISE) and molecular docking. ISE was employed to virtually screen 1.56 million molecules for docking interactions with PPARδ, resulting in the selection of 306 candidates for *in vitro* testing. Ultimately, 13 compounds were identified, with compound **18** (EC_50_ = 4 nM) (Figure 3) demonstrating highly selective agonism against PPARδ^145^.

This review described several natural products with anti-ageing activity and small molecule compounds showing potential for promoting collagen synthesis. However, natural products display notable limitations. Most natural products exhibit a range of biological activities, but the mechanisms by which they promote collagen synthesis often remain unclear. For instance, Esculetin demonstrates anti-inflammatory, anti-cancer and collagen synthesis-promoting effects. Furthermore, the extraction and purification processes for these natural products are complex, and their structural stability may be influenced by external factors. For example, terpenes are prone to oxidation, resulting in a reduction in their effective absorption. Additionally, some natural products with high molecular weights, such as ginsenoside Rb1, may encounter difficulties in crossing cell membranes, and their metabolites may possess differing biological activities. Moreover, while certain natural products may exhibit promising results *in vitro*, their efficacy may diminish in the *in vivo* studies.

This review also focuses on small molecule compounds targeting PPARδ. Despite their high target specificity, these compounds are not currently utilised in promoting collagen synthesis. Further in-depth investigations are needed to confirm the mechanism of its anti-skin ageing and promotion of collagen synthesis. Moreover, the clinical application of small molecule compounds requires stringent safety assessments and clinical trials.

## Conclusion

In recent years, increasing numbers of individuals have expressed concern about skin ageing. Research indicates that collagen is a critical component in maintaining skin elasticity and structural integrity. However, the ability of human fibroblasts to synthesise collagen diminishes, while collagen hydrolysis increases with ageing. This imbalance ultimately contributes to ageing manifestations such as roughness, laxity and wrinkles in the skin.

Collagen synthesis is primarily regulated by several signalling pathways, including the TGF-β/Smad, the PPARβ/δ, the JAK/STAT, the PI3K/Akt, integrin-related pathways and the pathways involving lysine hydroxylase and proline hydroxylase. These pathways represent potential therapeutic targets for developing anti-ageing treatments for the skin.

Currently, several natural products can enhance collagen synthesis by activating associated pathways, thereby exhibiting anti-skin ageing effects. For instance, esculetin promotes collagen synthesis by activating the PI3K/Akt pathway; santamarine and various flavonoid derivatives stimulate collagen synthesis by activating the TGF-β/Smad pathway; cinnamaldehyde enhances collagen synthesis by activating the upstream molecule IGF-I in the PI3K/Akt pathway; and ginsenoside Rb1, along with magnesium lithospermate B, promotes collagen synthesis via the PPARδ signalling pathway. However, there is a limited number of small molecule agents that effectively promote collagen synthesis for anti-skin ageing purposes. Among these, PPARδ agonists represent a class of small molecules with significant potential for anti-skin ageing, as they promote collagen synthesis by activating the PPARδ signalling pathway.

This review described the classification, structure and biosynthetic processes of collagen, as well as the target pathways that promote collagen synthesis. It also provided a detailed review of natural products and potential small molecule compounds with anti-skin ageing activity, including PPARδ agonists. The information presented is valuable for the advancement of anti-skin ageing drug development. Future research is anticipated to concentrate on elucidating the molecular mechanisms of skin ageing, thereby providing a theoretical foundation for the identification of small molecule drugs combating skin ageing. These efforts are expected to facilitate the development of highly effective, safe and reliable anti-skin ageing treatments.

## Abbreviations

AF-1: activation function-1
ALK5: activin receptor-like kinase 5
BMP: bone morphogenetic protein
Calf-1, Calf-2: calf domains 1 and 2
CCD: coiled-coil domain
CT: cytoplasmic tail
CTGF: connective tissue growth factor
DBD: DNA-binding domain
DGEA: aspartate-glycine-glutamate-alanine
ECM: extracellular matrix
EGF: epidermal growth factor
FGF-2: fibroblast growth factor 2
GABA: gamma-aminobutyric acid
GDF: growth and differentiation factor
HDF: human dermal fibroblast
HMC: human mesangial cell
HSC: hepatic stellate cell
IGF-I: insulin-like growth factor I
ISE: iterative stochastic elimination
JAK/STAT: Janus kinase/signal transducer and activator of transcription
LBD: ligand-binding domain
LD: linker domain
MIF: migration inhibitory factor
MMP: matrix metalloproteinase
mRNA: messenger RNA
ND: *N*-terminal domain
PDK1: 3-phosphoinositide-dependent protein kinase-1
PI3K/Akt: phosphoinositide 3-kinase/Akt
PIP2: phosphorylate phosphatidylinositol 4,5-bisphosphate
PIP3: producing phosphatidylinositol 3,4,5-trisphosphate
PPARβ/δ: peroxisome proliferator-activated receptors beta/delta
PPRE: PPAR response element
PSI: plexin-semaphorin-integrin
RPE: retinal pigment epithelium
RTK: receptor tyrosine kinase
RT-PCR: real-time polymerase chain reaction
RXR: retinoid X receptor
TAD: transcription activation domain
TGF-β: transforming growth factor-beta
TM: transmembrane
UV: ultraviolet
VEGF: vascular endothelial growth factor
Vps34: vacuolar protein sorting 34
α-SMA: α-smooth muscle actin
